# Shaping the Social: design of a settings-based intervention study to improve well-being and reduce smoking and dropout in Danish vocational schools

**DOI:** 10.1186/s12889-015-1936-6

**Published:** 2015-06-20

**Authors:** Susan Andersen, Janne Schurmann Tolstrup, Morten Hulvej Rod, Annette Kjær Ersbøll, Betina Bang Sørensen, Teresa Holmberg, Christoffer Johansen, Christiane Stock, Bjarne Laursen, Line Zinckernagel, Anne Louise Øllgaard, Liselotte Ingholt

**Affiliations:** Centre for Intervention Research in Health Promotion and Disease, National Institute of Public Health, University of Southern Denmark, Øster Farimagsgade 5A, DK-1353 Copenhagen, Denmark; National Institute of Public Health, University of Southern Denmark, Øster Farimagsgade 5A, DK-1353 Copenhagen, Denmark; The Danish Cancer Society Research Center, Strandboulevarden 49, DK-2100 Copenhagen, Denmark; Institute of Public Health, University of Southern Denmark, Niels Bohrs Vej 9, DK-6700 Esbjerg, Denmark

**Keywords:** Adolescent, Evaluation studies, Health promotion, Intervention studies, Smoking, Wellbeing, Social environment, Student dropouts, Vocational education

## Abstract

**Background:**

The social environment at schools is an important setting to promote educational attainment, and health and well-being of young people. However, within upper secondary education there is a need for evidence-based school intervention programmes. The Shaping the Social intervention is a comprehensive programme integrating social and educational activities to promote student well-being and reduce smoking and dropout in upper secondary vocational education. The evaluation design is reported here.

**Methods/design:**

The evaluation employed a non-randomised cluster controlled design, and schools were selected to either implement the intervention or continue with normal practice for comparison. In the baseline survey conducted 2011–2012, 2,329 students from four intervention schools and 3,371 students from six comparison schools answered a computer-based questionnaire during class, representing 73 % and 81 % of eligible students, and 22 % of all technical/agricultural vocational schools in Denmark. Follow-up assessment was conducted 10 weeks after baseline and at the same time teachers of the intervention classes answered a questionnaire about implementation. School dropout rates will be tracked via national education registers through a 2-year follow-up period.

**Discussion:**

Shaping the Social was designed to address that students at Danish vocational schools constitute a high risk population concerning health behaviour as well as school dropout by modifying the school environment, alongside developing appropriate evaluation strategies. To address difficulties in implementing settings-based interventions, as highlighted in prior research, the strategy was to involve intervention schools in the development of the intervention. Baseline differences will be included in the effectiveness analysis, so will the impact of likely mediators and moderators of the intervention.

**Trials registration:**

ISRCTN57822968. Date of registration: 16/01/2013

## Background

Schools are important settings for health promotion in young people [1–5]. Health education aiming to improve knowledge, develop skills and modifying norms is frequently used in schools, for example addressing substance use. However, such interventions often have disappointing results [6, 7]. In recent years there has been an increased recognition of how modifying the school social and/or physical environment can promote students’ health [8–10]. For example, a review found that class restructuring programs in which students spend more time with teachers were effective in decreasing school dropout [10]. Such a comprehensive approach is also known as the healthy school setting approach [11]. However, the evidence for this approach is relatively weak, in part because of difficulties related to both implementation and evaluation [12].

Several longitudinal studies have shown that harmful substance use predicts poor educational achievement [13, 14] and that educational failure is predictive of harmful substance use [15, 16]. Thus, a comprehensive perspective on both health risk behaviour and school dropout should be considered in intervention programmes aiming at promoting health and educational attainment.

In Denmark, approximately half of students drop out of vocational schools, which is a much higher rate compared to 18 % high school dropout [17]. The educational programme at vocational schools consist of a short basic course (typically 10–40 weeks) followed by a main course. Most dropout occurs during basic training. In addition, students more often engage in health risk behaviour compared to high school students [18]. For example, 23 % of vocational students and 9 % of high school students have tried drugs other than cannabis [18].

Ethnographic fieldwork performed at basic courses in Danish vocational schools indicates that vocational schools do not prioritise the development of social relations [19]. Students primarily work individually, and there is little time for social activities [19]. Students’ lack of social relationships in school can disrupt their educational pursuits. Young people who feel that they do not fit in at school are less likely to show up at school [1]. Previous research found that students who dropped out had low academic motivation, felt socially excluded, and did not have stable supportive adults [20]. In Danish vocational schools, smoking plays a central role in social interactions, and thus the students’ focus drifts away from achievement of professional skills [19]. This suggests that the school context enhances the use of cigarettes [21, 22]. Moreover, the relationship between academic achievement and substance use might be dynamic [23], so students with lower levels of academic achievement are more likely to engage in cigarette smoking.

In spite of the ample research evidence that points at the significance of the social environment for educational attainment, and the health and well-being of young people, little is known of specific intervention programmes that may be put to use in the context of vocational training. The aim of Shaping the Social was to develop and evaluate an intervention that promotes academic and social integration in vocational students to improve well-being, and reduce smoking and dropout in Danish vocational schools. Academic integration refers to students’ development of a strong affiliation with the school academic environment, including perceived fit with the educational programme and academic interactions with school staff, whereas social integration refers to development of a strong affiliation with the school social environment, including peer group interactions and informal contact with school staff [24].

We use a healthy setting approach. The healthy setting approach is criticized that it has a relatively weak evidence base for efficacy [12, 25]. This criticism should be seen in light of the fact that few studies focus on comprehensive programs and there are problems with both implementation and evaluation [12, 26]. Considering these limitations, there is a need for research on interventions with many components which in its design carefully take into account how initiatives are implemented and modified locally and what is an appropriate evaluation design. Therefore, this article aims to describe the evaluation strategies and response rates of Shaping the Social, a settings-based intervention study aimed to modify how the school environment influences well-being, smoking and dropout among students in upper secondary vocational education. In addition, we compare characteristics of the study population with the population from which it has been drawn in order to determine representativeness of the study sample.

## Methods

### Study design

Shaping the Social employed a non-randomised cluster controlled design with four intervention schools and six comparison schools. Comparison schools continued with their usual practice.

Intervention schools were selected by convenience sampling; the four schools involved in the development phase of the intervention agreed to become intervention schools. We chose this strategy in order to facilitate the implementation. The four intervention schools are located in major towns dispersed in Denmark and the schools are characterized as being large schools and offering a large range of technical or agricultural basic courses, for example carpenter and zookeeper. Characteristics of the intervention schools were used to select comparison schools among schools with the best match. However, a straight matched-pair design was not possible. Vocational schools in Denmark were ranked according to the following criteria: (1) at least one basic course offered for inclusion in the intervention group, (2) large school size (student population ≥ 800), (3) urban/suburban. Sixteen vocational schools were eligible. Subsequently, eight comparison schools were selected by geographic diversity. Letters explaining the study were sent to the school management, and schools expressing an interest (*n* = 7) were then visited individually. One school withdrew due to the time commitment entailed in the data collection. After selection of comparison schools, one intervention school wanted to include agricultural basic courses, which we accepted; however, the comparison schools were not selected on this criterion.

### Intervention content

Shaping the Social is a collection of intervention components to be used by school management and teachers in the intervention group; listed below. A detailed description of the intervention and the developmental work, including theories adapted and ethnographic fieldwork conducted for rationalising the intervention, is described elsewhere [19].

- A preliminary meeting at the school with a teacher, the students and their relatives
- Welcoming activities: Preparing classrooms, a welcome speech, an updated list of students, a round of person-to-person introductions, products of older students should be displayed and the students should begin working on an assignment relevant to their education, preferably in groups.
- Timetable with a clear description of course, time, place and clothing requirements
- Daily class meetings with mandatory participation for all students and their teachers. Coffee/the is served and the curriculum for the day is planned.
- Scheduled breaks: All students take breaks at the same time, and smoking is only allowed during the scheduled breaks
- Pleasant non-smoking place to hang out during breaks is set up
- Monthly events during school hours across sections (optional component)
- Open workshop with student access to school facilities outside school hours and a teacher present (optional component)

A staff pamphlet was developed with a description of the intervention components, and the intended immediate impacts of these components. During the study period, intervention schools had regularly contact with the research team, some departments more intensively because a formative process evaluation was conducted; description of the process evaluation itself is beyond the scope of this article.

**Fig. 1:**
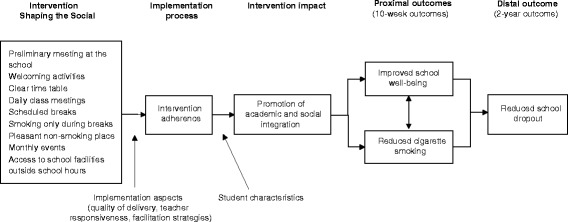
The hypothesised causal relationship between Shaping the Social intervention programme and proximal and distal outcomes

Figure 1 outlines the programme theory of Shaping the Social. Reduced school dropout is hypothesised to be caused by intervention efforts to improve student well-being and reduce the frequency of cigarette smoking during school hours. Intervention-induced improvements in student well-being and smoking are hypothesised to be caused by improved academic and social integration. Measuring these mediating processes should facilitate the understanding of short-term programme effects on student well-being and smoking. In addition, we measured whether the intervention was implemented as intended. Figure 1 also reflects our recognition that the magnitude of the intervention’s impact may depend on several moderating student characteristics.

### Setting

After nine compulsory years of primary and lower secondary school, almost all young people of Denmark choose to continue into the academically oriented upper secondary schools system, which is preparing students for tertiary education or into vocational education organized in separate schools spread out across large and medium sized cities. About 23% will choose vocational education [27]. Vocational education includes commercial, agricultural, technical, and social and healthcare programs leading to professions such as farmer, carpenter or web integrator. All programs are initiated by a school-based basic course lasting 20–25 weeks. The basic courses usually started four times a year and are flexible in duration depending on the student’s prior qualifications and ambitions. This means that some students within a class finish the basic course before others, and therefore a class is not a definite unit. Additionally, the high dropout rate implies that classes are merged during the basic course. We defined a class as students entering the same programme (e.g., carpenter) on the same date at the same address.

The vocational schools are organized into two levels. The upper level is the school with a management board. Next level is departments (e.g., construction) which are each headed by an inspector, one or more educational managers and an administration manager, and, typically, the departments have separate physical area and culture. In Shaping the Social, the upper analyze level will be these departments within schools (named college units).

### Participants

The study population consisted of students who attended the following basic courses (vocational clusters in brackets):

- Car mechanics
- Carpenter, bricklayer, painter or plumber (Construction)
- Electrician, data technician or frontline PC supporter (Electricity and information and computing technology (IT))
- Media graphic designer, graphic technician, web integrator or sign writer (Media production)
- Zookeeper, landscaper, farmer or greenhouse gardener (Agricultural)

Shaping the Social is designed to accommodate the fact that this was a heterogeneous population considerably more diverse than, for example, a typical Danish high school population.

### Data collection

The students answered a questionnaire within the first week of their basic course (T0) and at 10-week follow-up (T1). Teachers of the classes that were involved in the intervention answered a questionnaire about implementation at 10-week follow-up. School dropout rates will be tracked via national education registers [28] through a 2-year follow-up period (T2). Table 1 presents an overview of the collected data and the study time path. All residents in Denmark have an unique and permanent personal identification number, which allows individual-level linkage between nationwide registries [29]. In the baseline and 10-week follow-up questionnaire, the students were asked for their personal identification number in order to match the responses between the data collections and to follow up on individuals in registers.

**Table 1:** Collection of data and time path

	Instrument	Baseline	Follow-up	
		T0	T1	T2
			10 weeks	2 years
Personal characteristics				
Sex, age, ethnic origin, ethnic identity, parental education [44], family social class, distance to school, living arrangement, having children, quantitative and qualitative aspects of social relations [36], health and health behaviour [36], level of Danish reading and writing, level of prior schooling, prior academic achievement and academic self-efficacy [45]	Self-reported	X		
Primary outcomes				
Student well-being [33]	Self-reported		X	
Cigarette smoking [36]	Self-reported	X	X	
School dropout	Register data			X
Secondary outcomes				
Life satisfaction [39], physical and psychological symptoms [37], loneliness [38], alcohol consumption, use of cannabis and other illicit drugs [36]	Self-reported	X	X	
Immediate outcomes				
Indicators of well-conducted introduction period	Self-reported	X	X	
Indicators of classroom management [33]				
Degree of implementation				
Preliminary meeting at the school with a specialist teacher, the students and their relatives	Teacher reported		X	
Welcoming activities at first school day	Teacher reported		X	
Timetable with a clear description of course, time and clothing requirements	Teacher reported		X	
Class meeting every morning including beverages or food	Teacher reported		X	
Scheduled breaks	Teacher reported		X	
Created a place for hanging out during breaks	Teacher reported		X	
Monthly events during school hours organised across sections, and followed by an open café	Teacher reported		X	
Access to school facilities outside school hours and a member of staff is present	Teacher reported		X	

We developed the student questionnaires with items based on Danish population surveys, and on validated scales when possible. The questionnaires were subject to an expert hearing followed by pilot testing among students (*n* = 117) in three vocational schools (not included in the intervention study). Self-developed items, especially, were giving attention. We kept the questionnaires as short as possible in order to increase the response rate [30].

Data collections were performed during class and took approximately 20 min. Baseline assessment was not possible before school start, because school records of students were incomplete. Moreover, we chose to collect data during school time because the student population included socio-demographically diverse subgroups and a predominately male and young population, which are characteristics that can predict non-response [31, 32]. Questionnaires were web-based with an audio voice-over so the students heard questions through headphones while they appeared on screen. Follow-up of non-respondents in the 10-week-follow-up assessment was by electronic mail, mobile text message and a mailed letter.

Baseline data collection started in October 2011 and ended in October 2012, and comprised five samples of basic courses (autumn 2011, winter 2012, spring 2012, summer 2012 and autumn 2012).

### Outcome measures

Our primary outcomes were student well-being at school, smoking and school dropout.

*Student well-being* was conceptualised as positive student interpersonal relations, positive student-teacher relations, school connectedness and positive valuing of the profession, and was measured on items developed for the Danish version of the Health Behaviour in School-aged Children (HBSC) surveys [33] and new items. The HBSC measures is designed to capture a broad conception of student well-being at school and have been piloted for international use in samples of 13- and 15-year-old students and have shown adequate validity and reliability [34]. Student interpersonal relations were measured by a 5-item HBSC classmate support scale (e.g., ‘the students in my class enjoy spending time together’). Student-teacher relations were measured on a 3-item HBSC scale (e.g., ‘I feel that my teachers accept me as I am’). School connectedness was measured by three HBSC items (e.g., ‘I feel I belong at my school’) [35]. Valuing of the profession was measured by four Shaping the Social items (e.g., ‘I am proud of my profession’).

*Cigarette smoking* was measured by daily smoking status (yes, no) and current number of cigarettes smoked per day [36].

*School dropout* will be tracked by national education registers from Statistics Denmark. The students will be categorised into one of the following categories: (a) completed the basic course, (b) still registered in the basic course or changed to another upper secondary education, (c) dropped out from basic course and has not attained another upper secondary education.

For secondary outcomes we assessed well-being in general by physical and psychological symptoms measured on the HBSC Symptom Checklist (e.g., difficulty sleeping) [37], loneliness measured by one item [38], and life satisfaction measured on the Cantril Ladder Scale [39] with ten positions ranging from zero (the worst possible life) to ten (the best possible life). For unintended effects we assessed alcohol consumption and use of cannabis and other illicit drugs than cannabis (e.g., cocaine) [36]. We measured alcohol and drug consumption as side-effects but it is difficult to predict whether they will occur as unintended adverse or positive effects. Increased school well-being has a positive impact on alcohol and drug use [40]. However, the literature also indicate that young people who have an active social life have more drunkenness-oriented drinking patterns and are experimenting more with drugs [41].

### Measures on the causal pathways

#### Intervention implementation

Process data were collected to determine the implementation degree [42]. For each class, the teachers completed a questionnaire, which addressed implementation questions measured in two ways: (i) Have essential prescribed intervention components been delivered (e.g., the students had to work in groups on an assignment relevant to their education) with response options “yes”, “no” and “don’t know”; (ii) Have the components been delivered in the intended way (e.g., the students were made to feel welcome) measured on a 10-point Likert-type scale (1 = strongly disagree to 10 = strongly agree). Strategies to facilitate the implementation were rated by the level of feedback provided to the teachers and managers (high, medium, low). Intervention implementation was also investigated as part of class observations and interviews with school staff and students, which were elements of the process evaluation carried out by researchers (LI and BBS) in a sample of classes over the course of the study. Conclusions regarding implementation degree will be compared to these qualitative findings.

#### Immediate intervention impact

To address the immediate impact from the intervention, selected indicators regarded the introduction period (e.g., student felt welcome on the first day) and classroom management (e.g., our teachers intervene if we get distracted) were included in the student surveys.

### Personal characteristics

In addition to the outcome variables, process variables and immediate variables, the following variables were collected at baseline (see Table 1): Age, ethnic origin (determined by the place of birth of the mother, substituted by the place of birth of the father if maternal birthplace was missing and substituted by the place of birth of the student if both maternal and paternal birthplaces were missing), ethnic identity [43], parental education level [44], family social class with two items on father’s and mother’s employment status and two items on father’s and mother’s occupation, and minutes spent travelling to the school. To measure quantitative aspects of social relations, we used items from the Danish National Health Survey studies [36] to ask about how often the students have contact to friends, the students’ living situation and having children. To measure qualitative aspects of social relations, we included social support measured as the extent to which the students had someone to talk to when they had problems or needed support [36] and a HBSC item about frequency of feeling left out. To measure health behavior and health [36], we included cigarette smoking, alcohol consumption, cannabis use and use of illicit drugs other than cannabis, self-confidence and self-rated health. To measure the students’ educational background, we included academic self-efficacy [45], self-evaluated school performance, assessment of Danish reading and writing (separately) and overall prior school satisfaction

### Sample size

Sample-size calculation was performed to determine the number of classes needed in order to be able to detect a difference between the intervention and comparison group with respect to student well-being, smoking and school dropout [46]. From a nationwide student satisfaction survey in 2010 at Danish vocational schools, we selected measures on daily smoking (yes, no), consideration of dropping out (yes, no), and two indicators on student well-being: solidarity among students (yes, no), and a positive welcoming atmosphere created by the school (yes, no). Overall, 41 % were daily smokers, 19 % considered dropping out, 17 % reported a lack of student solidarity, and 21 % reported a lack of a positive welcoming atmosphere. We estimated the intra-class correlation to 0.006 for welcoming atmosphere, 0.05 for student solidarity and smoking, and 0.016 for dropout considerations. With a desired power of 80 %, a 5 % significance level, a two-sided test, and an expected 20 % reduction for each outcome in the intervention group, the minimum number of classes in intervention and comparison groups was calculated as ranging from 60 to 110. To be conservative, we chose the highest number of clusters (i.e., classes, *n* = 110). Another necessary assumption for the power calculation was that each class had an average of 20 students. Allowing for 20 % non-respondents, a total of about 5,280 students needed to be included.

### Evaluation methods

Initially, we will conduct scale validation of the student well-being outcome measure, which we hypothesise to be a four-factor model. Intervention effects will be evaluated by multilevel regression methods, specifically, a multilevel regression analysis accounting for the hierarchical data structure with students nested within classes in which the intervention was implemented, and classes nested within college units. We will use mediation analyses to explore the extent to which any intervention effects are mediated by changes in the measured immediate intervention impacts. The analyses will be conducted for the total study population. The smoking analysis will be repeated whereby students are analyzed according to their baseline smoking status. Analysis of subgroups will be conducted defined by age, parental socioeconomic status and basic course. To assess the stability of the available case analyses, handling of missing data will be conducted and analysis of loss to follow-up will indicate which variables to include.

### Study status

The study is ongoing and the investigators are at present analysing data.

### Participant flow

**Fig. 2:**
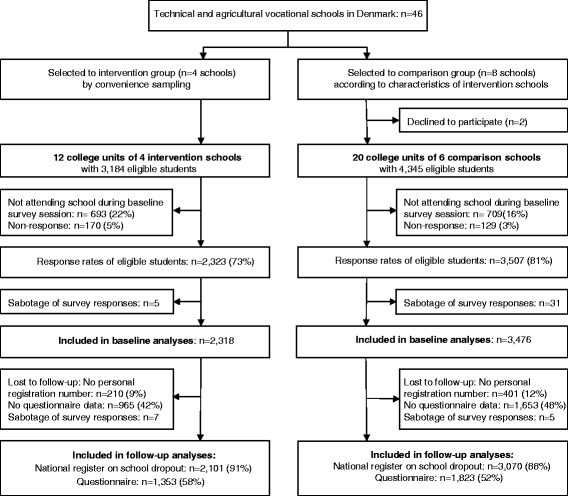
Flow diagram of recruitment and participation in the Shaping the Social study

In total, we included 32 college units of 10 large and diverse technical and agricultural vocational schools; a sample of approximately 22 % of all Danish technical and agricultural vocational schools. Figure 2 shows the flow of students through the recruitment and analysis phase. The baseline survey resulted in responses from 2,329 students from four intervention schools and 3,371 students from six comparison schools, representing 73 % and 81 %, respectively, of the students assumed to be eligible. The true rate is likely to be higher because, as is typical, a number of students never started, despite enrolment on the class list, and were thus not eligible for inclusion. The majority (95 % and 97 %, respectively) who were in school on the day of data collection did respond to the questionnaire. Forty-eight students were excluded because of two or more errors identified in the data control (e.g., they reported smoking ≥ 70 cigarettes per day). The response rates for the follow-up questionnaire survey were 58 % and 52 %, respectively. The response rates of reporting the personal identification number (necessary for linkage with the education register to obtain dropout at the second follow-up) were 91 % and 88 %, respectively.

### Representativeness of the study sample

Convenience samples may be biased if the sample not fully represent the population from which it has been drawn, thus the frequencies observed in the convenience sample cannot be generalised to the larger population. Based on data from nationwide registers we can determine the representativeness of the study sample. From Statistics Denmark, we have access to population data on sex, age, prior education and ethnicity of all students entering vocational education for the period of 1.10.2011 until 30.09.2012 [47]. In total, 22511 students did entering vocational education of the technical and agricultural vocational clusters comprised the study base. Formulas proposed by Sousa et al. (2004) [48] were used to calculate the expected counts and range of average variability. Data are shown in Table 2. For example, 18311 (81.3 %) students of the research setting population were male, whereas 4703 (81.2 %) students of the study sample were male. Under a normal approximation, 95 % of the study sample’s count of males was expected to fall between 4663 to 4763. Thus, the study sample’s count of males was in the acceptable range. Overall, analysis of percentages as well as expected counts and average variability indicated that the study sample and the study base were almost identical; indicating that the study sample was representative of the population in relation to sex, age, prior education and ethnicity.

**Table 2:** Demographic characteristics of the research setting population and the study sample, and expected sample counts

	Population of the research setting (*n* = 22511)^a^		Study sample (*n* = 5794)^b^		Expected sample counts^d^	
	n	%	n	%	n	95 % CI
Sex						
Male	18311	81.3	4703	81.2	4713.0	4662.9 − 4763.1
Female	4200	18.7	1091	18.8	1081.0	1030.9 − 1131.1
Age groups, years						
15-18	10575	47.0	2700	46.6	2719.5	2655.3 − 2783.7
19-24	8262	36.7	2130	36.8	2124.7	2062.7 − 2186.7
>24	3674	16.3	959	16.6	944.8	897.3 − 992.3
Prior education						
Completed only elementary school or less	17520	81.6	4561	80.6	4615.4	4565.7 − 4665.1
Completed an education above elementary school^c^	3954	18.4	1096	19.3	1041.6	991.9 − 1091.4
Ethnicity						
Danish origin	20019	89.8	5105	89.2	5141.5	5102.6 − 5180.4
Immigrants/descendants	2272	10.2	620	10.8	583.5	544.6 − 622.4

^a^1-10-2011 to 30-9-2012; Statistics Denmark

^b^Self-reported

^c^Vocational school, high school, or higher education

^d^Calculated by formulas proposed by Sousa et al. (2004) [47]

## Discussion

In this article the study design of a settings-based intervention targeting well-being, smoking and dropout among students in upper secondary vocational education is described; in a sample of approximately 22 % of all Danish technical and agricultural vocational schools. In addition, the representativeness of the convenience sample is determined. Because school dropout and health risk behavior represent major problems among students attending vocational education in Denmark and many other countries [49], there is a need for evidence-based intervention programs in the vocational school setting. Furthermore, students attending vocational education have less favourable health practices than the general population, and it is a more heterogeneous population than students attending general upper secondary education. The design of this intervention study takes account of these issues by adopting a comprehensive perspective on both health behaviour and the social environment at school.

### Study strengths and limitations

The strength of Shaping the Social is the relatively high response rate at the baseline survey, which may have been due to our efforts to collect data during school hours. Dropping out of school is a condition of the vocational school setting, which adversely affected the retention rates. However, the high submission of the personal identification number in the baseline survey makes it possible for us to link data to education registers at an individual level. Thus, we will be able to determine the intervention’s long-term effect on educational attainment.

The aspect that creates the intervention’s potential success (its settings-based comprehensiveness) also creates potential difficulties in analysing its effectiveness. The variety of factors that contribute e.g., to student well-being makes it challenging to clearly distinguish between successful and unsuccessful characteristics of the programme. The intervention’s comprehensiveness (establishment of new school practices and re-shaping existing practices) rules out a highly standardized intervention. This is addressed by collecting and analysing teacher data with respect to the school practices [42]. However, it proved infeasible to collect teacher data with respect to intervention components at the comparison schools. This may led to underestimation of effects, if the existing practices at comparison schools are similar to the intervention.

A second limitation is the lack of random allocation of the intervention between schools, which may introduce selection bias. However, analysis of the study sample and the research setting population showed that the study sample was representative of the population. Randomisation is not always possible when conducting school-based interventions [50]. First, there are a limited number of Danish vocational schools. Second, the schools involved in the development phase became intervention schools. This was necessary because the development phase facilitated the implementation, with time spent on internal organisation and integrating the intervention into the pre-existing school practices [51, 52]. We selected comparison schools in order to balance groups on important variables, such as type of basic course. Nevertheless, one basic course was included in the intervention group after selection of comparison schools. Whether unbalanced student characteristic impact outcomes will be examined and potentially controlled for in the analyses.

A third limitation is the time frame for baseline data collection. One substantially important design choice for the study was to collect baseline data during class in the first days of school – i.e., after the onset of the intervention – and this may contribute to underestimation of effects. When interpreting the results of the effect evaluation, the time frame for baseline data will be taken into account in the analyses.

## Conclusions

Research on the effectiveness of interventions that incorporate a perspective on health behaviour and the social environment for students at vocational schools are of substantial importance. We have demonstrated evaluation strategies of such an intervention, developed to address the school setting rather than individuals, including considerations of implementation and analysis.

### Ethical issues

The study was carried out in accordance with current Danish rules of ethics and legislature and has been approved by the Danish Data Protection Agency, 8 August 2011, record number 2011-54-1265. The National Committee on Health Research Ethics concluded that formal ethics approval was not required because no human biological material was sampled. There is no formal institution for ethical assessment and approval of register- and questionnaire-based population studies in Denmark.

### Consent procedure

When schools were invited to participate, written information was send to the school targeting the school management explaining the implications of participation in the study and we received approval of the student surveys. The study was introduced to students as a study about well-being, health behaviour and health with focus on preventing school dropout in all students. The students were informed that participation was voluntary, that their information would be used for research purposes only and treated confidentially and of the possibility of withdrawing during the study. A few students (1.7 %) were aged 15; otherwise the students were aged 16 or older. Based on Danish legislation and ethical constraints, young people aged 15 or older can make an independent decision to participation in surveys without parental consent [53].
